# Myogenous temporomandibular disorders and salivary markers of oxidative stress—A cross‐sectional study

**DOI:** 10.1111/joor.13100

**Published:** 2020-10-05

**Authors:** Víctor Ignacio Madariaga, Hajer Jasim, Bijar Ghafouri, Malin Ernberg

**Affiliations:** ^1^ Division of Oral Diagnostics and Rehabilitation Department of Dental Medicine Karolinska Institutet and Scandinavian Center for Orofacial Neurosciences (SCON) Huddinge Sweden; ^2^ Pain and Rehabilitation Centre, and Department of Health, Medicine and Caring Sciences Linköping University Linköping Sweden

**Keywords:** antioxidants, craniomandibular disorders, myalgia, myofascial pain syndromes, oxidative stress, saliva

## Abstract

**Background:**

The clinical care of chronic pain requires personalised understanding of the mechanisms involved. Temporomandibular disorders (TMD) are the most common chronic orofacial pain conditions, and oxidative stress has been proposed to be implicated in their pathophysiology, especially in arthrogenous TMD. However, few studies have explored oxidative stress in myogenous TMD (TMDM).

**Objective:**

The aims of this study were to compare the salivary oxidative stress profiles between individuals with TMDM and healthy controls, and to explore associations of these markers with clinical characteristics.

**Methodology:**

Saliva samples were collected from 39 individuals with TMDM and 37 age and sex‐matched healthy volunteers. Psychological stress levels and clinical characteristics were assessed in all participants. The samples were analysed for total oxidant status (TOS), total antioxidative capacity (TAC) and superoxide dismutase activity (SODa). Comparisons between groups were performed using parametric and non‐parametric tests depending on data distribution.

**Results:**

Psychological stress was higher in TMDM compared to controls (*P* < .001). TAC levels were significantly higher (*P* < .05) whereas TOS levels were significantly lower (*P* < .05) in TMDM compared to controls. There were no differences in SODa levels between groups and no correlations were found between clinical characteristics and oxidative stress markers.

**Conclusion:**

Individuals with TMDM showed higher levels of antioxidative markers, but lower levels of oxidative markers. These results can be explained in part by chronicity and adaptation to the disease and other factors, such as psychological stress. Longitudinal studies must be conducted to clarify the role of oxidative stress in TMDM.

## 1 BACKGROUND

The clinical care of chronic pain disorders has evolved to a point where multiple biopsychosocial approaches coexist in order to manage a person who suffers.^1^ But this requires an understanding of the different components of pain in an individual, including the pathophysiological mechanisms—which is essential for personalised treatment.^1^, ^2^ In temporomandibular disorders (TMD), these are not yet well characterised.

TMD are a group of chronic conditions comprising ‘pain and dysfunction of the masticatory muscles and temporomandibular joints (TMJ)’,^3^ associated with high general and psychological disease burden and impaired quality of life.^3^, ^4^ Symptoms include, for example, pain in the TMJ and masticatory muscles, limited jaw movement and articular noises.^3^, ^4^ Among TMD, myogenous TMD (TMDM) are the most common conditions with a prevalence of 45.3% of the cases.^5^ According to the Diagnostic Criteria for TMD (DC/TMD), TMDM can be subdivided into myalgia and myofascial pain depending on whether pain is localised during palpation (myalgia) or spreads within (myofascial pain) or beyond (myofascial pain with referral) the palpated muscular territory.^3^, ^6^

Much has been researched about TMDM, but the pathophysiology is not completely understood; they are considered to have a multifactorial origin.^3^ Central and peripheral sensitisation together with impairment of the descending inhibitory pathways in the central nervous system seem to play a relevant role in their development.^3^, ^7^ Studies have associated the initiation and/or perpetuation of TMDM with muscle ischaemia (due to repetitive contraction) and release of inflammatory mediators.^3^

Oxidative stress is an imbalance between the production of reactive oxygen species (ROS) and counteracting antioxidant mechanisms.^8^ Studies suggest that oxidative stress can result from inflammatory processes and psychological alterations (such as psychological stress). Both mechanisms are associated with the development of TMD, which may have a pathophysiological relationship with oxidative stress as well.^9^, ^10^, ^11^

Researchers have proposed that this relationship between ROS and disease could be useful to identify sub‐populations that can be managed in the context of oxidative stress.^12^ Accordingly, several products of cell damage by ROS have been utilised as markers of this process.^9^ The overall activity of both oxidants and antioxidants can be measured as the total oxidant status (TOS) and the total antioxidant capacity (TAC); these have been used in clinical and animal studies to address the activity of such molecules as a whole.^13^, ^14^

Concerning oxidative stress markers in TMD, previous studies have reported that the saliva levels of TAC and TOS differ from healthy controls.^13^, ^15^, ^16^ However, these included mainly individuals with arthrogenous disorders, and none focused on TMDM. Specifically studying TMDM might give some answers about their pathophysiology and whether management strategies should consider oxidative stress levels in the future. Therefore, the first question addressed in this article was given as: is there a difference in the oxidative stress profile of individuals with TMDM compared to healthy controls? Moreover, oxidative stress as a mechanism of disease could relate to specific features of TMDM. Hence, the second question was given as: are oxidative stress markers related to particular clinical or psychosocial characteristics in TMDM?

We hypothesised that if oxidative stress is involved in the pathophysiology of TMDM their salivary levels would be altered in individuals with these conditions. We also hypothesised that these differences would relate to specific characteristics of these disorders (ie pain, jaw function or psychological stress). Therefore, the aims of this study were to compare the salivary oxidative stress profiles between individuals with TMDM and healthy controls, and to explore associations of these markers with clinical characteristics.

## 2 METHODOLOGY

### 2.1 Study design and ethical considerations

This cross‐sectional investigation was a sub‐study from another project conducted at the Department of Dental Medicine at Karolinska Institutet, Huddinge, Sweden.^17^ The study was performed in agreement with the Helsinki declaration and was approved by the Stockholm Ethical Board (2014/17‐31/3).

### 2.2 Participants

**FIGURE 1:**
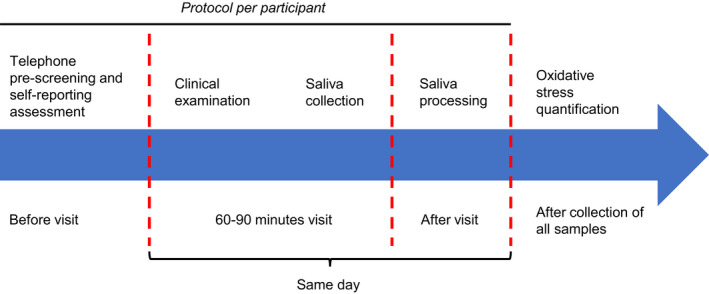
Experimental timeline. Each potential participant was asked to respond a questionnaire by telephone. Those who met the requirements were invited for a clinical assessment, and during that session, if they met the inclusion criteria, saliva samples were collected and processed on the same day. At the end of the recruitment period, stored saliva samples were analysed all together [Colour figure can be viewed at wileyonlinelibrary.com]

For a full description of the methodology, see Jasim et al.^18^ In brief, from June 2017 to April 2019, individuals with TMDM were recruited from patients referred to the Specialist Clinic for Orofacial Pain and Jaw function, University Dental Clinic, Karolinska Institutet, Huddinge, Sweden; together with healthy and pain‐free volunteers who responded to public advertisement or were undergraduate dental students at the same institution. Sample sizes from the original study were calculated based on data from similar studies at the department, considering two groups, a difference of 1.5 SD, a statistical power of 0.8 and statistical significance of *P* < .05, which yielded a sample size of 37 in each group. Once a participant was recruited, a telephone questionnaire was used for the pre‐screening and collection of self‐reporting data (eg DC/TMD axis II^6^) from the potential participants. Those who met the pre‐screening requirements were invited for an assessment visit and were instructed to not take pain killers or non‐steroidal anti‐inflammatory drugs (NSAIDs) at least for 24 hours before; to not have eaten and brushed their teeth in two hours before; and to avoid the consumption of alcohol and food rich in tryptophan for at least 24 hours before. During the same visit, a clinical examination was performed, an informed consent was signed, and saliva samples were collected from those who met the inclusion criteria (the whole process is illustrated in Figure 1). In the original study, 39 individuals with TMDM and 39 healthy controls were included; however, saliva samples from two of the controls were insufficient to perform the oxidative stress’ analyses, as these were done posterior. Therefore, samples from 39 individuals with TMDM and 37 controls were included.

### 2.3 Inclusion and exclusion criteria

The inclusion criteria for TMDM were given as: a diagnosis of either myalgia or myofascial pain (with or without referral) according to the DC/TMD,^6^ a pain duration of at least three months, and an average pain intensity of ≥3/10 on a 0‐10 numeric rating scale (NRS) during the last 30 days. Other DC/TMD diagnoses were allowed only if a TMDM was the main complaint. The inclusion criteria for healthy volunteers were given as: self‐reported healthy status, and no evidence of disease or current pain. Moreover, the age range of inclusion for both groups was 18‐45 years.

The general exclusion criteria for all participants were given as: any condition that could influence pain sensitivity, neurological disorders, pain of dental origin, pregnancy or lactation, and high blood pressure. Participants were also excluded when taking medications that could interfere with analysis, pain sensitivity or pain perception such as anticoagulants, analgesics or antidepressants during the last two weeks before the study. Other exclusion criteria were given as: smoking or the use of snuff (oral tobacco), and factors that could influence saliva collection and composition, such as hypo‐salivation, salivary gland diseases, poor oral hygiene, several missing teeth, extensive prosthodontics rehabilitations, oral diseases and mucosal lesions. No exclusion was considered regarding ethnic background.

### 2.4 Clinical examination and questionnaires

During the visit, participants underwent a general clinical dental examination and were further examined according to DC/TMD axis I by one trained researcher (HJ) calibrated to a reference standard examiner (ME).^6^

From the DC/TMD axis II questionnaire (previously performed), the following variables were extracted to contrast with oxidative stress markers in the saliva. As a general health parameter, self‐reported body mass index (BMI) measured in kilograms per square‐metre (kg/m^2^) was assessed. Current pain intensity was assessed with NRS ranging from zero to ten (zero being no pain and ten being the most intense pain experience as imaginable by the participant) and the characteristic pain intensity (CPI), that is, the average of the current, and the worst as well as average pain intensity felt in the last 30 days (NRS) multiplicated by ten was calculated to yield a score from zero to hundred. Additionally, the participants were asked about their pain duration (years). The Jaw Functional Limitation Scale (JFLS) score (mean global score of 1.74 and standard error of 0.11 for chronic TMD),^19^ and the maximal unassisted opening (MUO) measured with a ruler in millimetres (mm) were evaluated as measures of jaw function. For the evoked pain sensation, pressure pain threshold (PPT) was recorded utilising an electronic algometer (Somedic Sales AB, Hörby, Sweden) pressed against the skin area corresponding to the masseter muscle. The PPT registration was obtained as the averaged minimum force in kilopascal (kPa) at which the participant felt the slightest sensation of pain in three consecutive applications. As a psychosocial characteristic of interest, the psychological stress level was assessed with the 10‐item Perceived Stress Scale (PSS‐10) (scale from zero to forty, categorising low, moderate or high perceived stress with upper limits of thirteen, twenty‐six and forty points, respectively).^20^, ^21^, ^22^

### 2.5 Saliva sample collection and processing

After the clinical examination, if the participants met the requirements, whole stimulated saliva was collected.^18^ All samples were obtained at the same time range, between 7:30 am and 12:00 pm. In brief, the participants were instructed to rinse their mouth during 30 seconds with distilled deionised water to remove remains of citric acid. Consecutively, the participants had to chew a block of paraffin gum (Orion Diagnostica, Finland), until it became smooth and flexible for one minute. The participants were instructed to swallow the saliva present in the mouth, and then, new saliva was collected in a polypropylene tube coated with protease inhibitor (Sigma Aldrich v/v 1:500) until a volume of 5 mL was reached. Finally, time to fill in the tube was measured to assess salivary flow rate.

Right after the collection of the samples, the samples were centrifuged at 2500 *g* for 15 minutes at room temperature to remove debris. The saliva supernatants were aliquoted in low binding 0.5 mL Eppendorf vials and stored at −70°C until analysis (full protocol in Jasim et al^18^).

### 2.6 Quantification of markers for oxidative stress

Oxidative stress was measured in terms of oxidant and antioxidant activity, since this might give a better image of functional balance in the context of oxidative stress.^23^ The analyses of these markers were performed from November 2019 to June 2020 using commercial kits. All analyses were blinded and performed by a researcher who did not participate in sample collection (VIM). TOS was measured in µmol of H_2_O_2_ equivalents per litre (µmol H_2_O_2_ Equiv./L) using the Total Oxidant Status Assay (Rel Assay Diagnostics, Gaziantep, Turkey) (limit of detection (LOD): 0.2‐80 µmol H_2_O_2_ Equiv./L). The level of antioxidation was evaluated as TAC in µmol of Trolox equivalents/L (µmol‐TE/L) using a Total Antioxidant Capacity Assay, ab65329 (Abcam, Cambridge, United Kingdom) (LOD: unspecified). Additionally, extracellular SOD activity (SODa) was measured utilising the Superoxide Dismutase Activity Assay Kit, ab65354 (Abcam, Cambridge, United Kingdom) (LOD: >0.1 U/mL of SOD activity), as the percentage of inhibition (% inhibition) of the enzyme WST‐1. All tests were performed following the protocol established by the manufacturers.

### 2.7 Statistical analysis

The background characteristics of the participants are presented with descriptive statistics mean and standard deviation (SD) or median and interquartile range (IQR). Frequencies between males and females were compared using Chi‐square test. All continuous variables were checked for normality by visual analysis of the histogram and the Q‐Q plot, and statistically by Shapiro‐Wilk test, skewness value (score between −1.0 and 1.0) and skewness z‐test (|z‐score| < 1.96) in each group. Comparisons were made between groups using Mann‐Whitney *U* test when data were abnormally distributed and Student's or Welch's *t* test when data were normally distributed. Correlation assessments for all the included variables were performed using Spearman's correlation coefficient. A *P*‐value < .05 was considered as statistically significant. Missing data were not considered in the statistical evaluations. For the correction of multiple comparisons, Hochberg's adjustment was done utilising R (version 3.6.3, R Core Team, 2020) and RStudio (version 1.2.5033, RStudio Team, 2019), considering markers of oxidative stress as the independent variables. The statistical software Jamovi (version 1.2.18, Jamovi Team, 2020) was utilised for all the analyses.

## 3 RESULTS

### 3.1 Participants data

Demographics and clinical data are presented in Table 1. Participants were mostly born in Sweden and mainly women similarly distributed among patients and controls. Apart from a main diagnosis of TMDM, 23 individuals (58.97%) had arthralgia, 29 (74.4%) had headache attributed to TMD, 10 (25.6%) had disc displacement with reduction, and 2 (5.1%) had degenerative joint disease. There was no statistically significant difference in the median age between TMDM and controls. In the TMDM subgroups, individuals with myalgia and myofascial pain did not differ in sex and country distributions, or age. Neither were there any significant differences in BMI between TMDM and controls, nor between TMDM subgroups. Psychological stress levels (PSS‐10) were significantly higher in TMDM than controls, but did not differ significantly between TMDM subgroups.

**TABLE 1:** Comparison between demographic and baseline characteristics of individuals with myogenous temporomandibular disorders (TMDM) and healthy controls as well as TMDM subgroups myalgia and myofascial pain with and without referral (MFP)

	TMDM n = 39	Healthy n = 37	*P*‐value	Myalgia n = 14	MFP n = 25	*P*‐value
Demographics						
Age (y)	27.0 (8.2)	28.2 (10.7)	0.743	23.9 (9.6)	27.2 (7.7)	0.334
Sex (% women)	82.1	81.1	0.913	71.4	88.0	0.196
Born in Sweden (%)	71.8	67.6	0.688	85.7	64.0	0.148
General health						
BMI (kg/m^2^)	23.7 ± 3.9	22.7 ± 3.3	0.254	22.6 ± 3.3	24.8 ± 3.1	0.240
PSS‐10 (0‐40)	17.0 (11.0)	10.0 (9.0)	<0.001	13.0 (10.1)	19.0 (7.0)	0.061
Spontaneous pain						
Duration (y)	5.0 (5.8)	0	<0.001	4.0 (6.3)	5.0 (5.0)	0.607
Current pain (0‐10)	4.0 (2.0)	0	<0.001	4.0 (2.0)	5.0 (2.0)	0.202
CPI (0‐100)	60.0 (20.0)	0	<0.001	53.0 (18.2)	63.0 (17.0)	0.146
Evoked pain						
PPT masseter (kPa)	180 ± 56.3	268 ± 71.5	<0.001	226 (53)	157 (40)	<0.001
Jaw function						
MUO (mm)	52.5 ± 6.4	58.1 ± 5.65	<0.001	53.0 (9.0)	52.0 (7.0)	0.191
JFLS (0‐10)	1.15 (1.7)	0	<0.001	0.50 (1.8)	1.6 (1.4)	0.055

Note

Results are presented as median (IQR) or mean ± SD *P*‐value < 0.05 as statistically significant (bold).

Abbreviations: BMI, body mass index; CPI, characteristic pain intensity; IQR, interquartile range; JFLS, Jaw Functional Limitation Scale; MUO, maximal unassisted opening; PPT, pressure pain threshold; PSS, Perceived Stress Scale; SD, standard deviation.

As expected, pain duration, current pain and CPI were significantly higher in TMDM than controls, but did not differ significantly between TMDM subgroups. PPT over the masseter was significantly lower in the TMDM group compared to healthy controls, and also lower in the myofascial pain group compared to the myalgia group.

The TMDM group had significantly lower MUO and higher JFLS score compared to the healthy controls. The TMDM subgroups did not differ significantly in MUO, or JFLS score.

### 3.2 Oxidative stress

**FIGURE 2:**
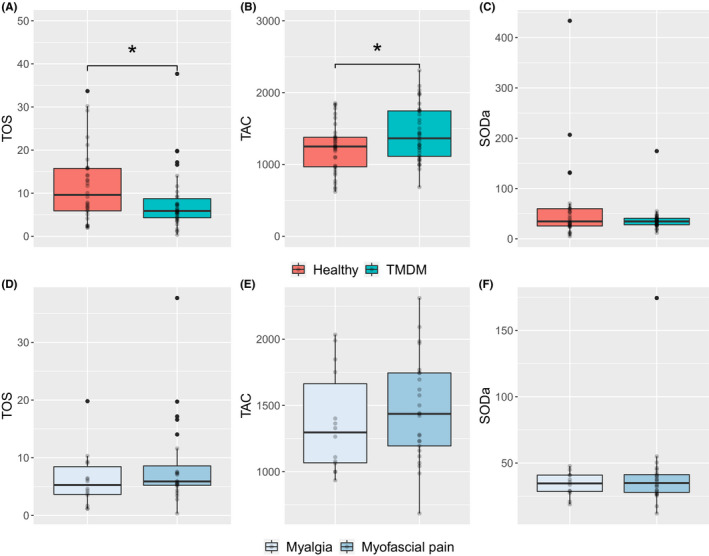
Comparison of salivary oxidative stress markers. (A) TOS (µmol H_2_O_2_ Equiv./L), (B) TAC (µmol‐TE/L) and (C) SODa (% inhibition) were compared between individuals with myogenous temporomandibular disorders (TMDM) and healthy subjects. Additionally, (D) TOS, (E) TAC and (F) SODa were compared between TMDM subgroups (myofascial pain and myalgia). Statistical analyses were performed using Mann‐Whitney U test. *= statistically significant (*P* < .05). SODa, Extracellular superoxide dismutase activity; TAC, total antioxidative capacity; TOS, Total oxidant status

As Figure 2 shows, the median levels of TOS were significantly lower in the TMDM group compared to the healthy controls (Figure 2A), but there was no significant difference between individuals with myofascial pain and myalgia (Figure 2D). In contrast, the TAC levels were significantly higher in TMDM compared to controls (Figure 2B). The TAC levels in the myofascial pain group were not significantly different compared to the myalgia group (Figure 2E). Neither was there any significant differences in SODa between TMDM and controls (Figure 2C), nor between TMDM subgroups (Figure 2F). Since some of the values obtained during the TOS assessment were out of the LOD, the results from one individual with myofascial pain and three controls were excluded from this analysis. Regarding the SODa tests, the results from six controls, one individual with myofascial pain and two with myalgia were excluded because the absorbance values were higher than the reference values (which is not allowed for the calculation of antioxidative activity).

To assess whether arthralgia had an influence on oxidative stress in TMDM, the levels of the respective markers were compared between individuals with or without such diagnosis. No statistically significant differences were found for TOS (*P* = .123), TAC (*P* = .165) or SODa (*P* = .056).

### 3.3 Correlation analyses

Correlation analyses were performed between clinical characteristics and markers of oxidative stress (TOS, TAC and SODa) in TMDM (Table 2). No significant correlations were found between any of these parameters.

**TABLE 2:** Correlation analysis between markers of oxidative stress and clinical or demographic characteristics of individuals with myogenous temporomandibular disorders

	TOS		TAC		SODa	
	*ρ*	*P*‐value	*ρ*	*P*‐value	*ρ*	*P*‐value
Demographics						
Age	‐0.119	0.477	0.188	0.477	‐0.187	0.477
General health						
BMI	0.298	0.261	0.216	0.440	0.109	0.560
PSS	0.186	0.528	0.037	0.822	‐0.191	0.528
Spontaneous pain						
Duration (y)	0.062	0.714	‐0.160	0.662	‐0.212	0.642
Current pain	0.136	0.908	‐0.124	0.908	‐0.012	0.943
CPI	0.197	0.470	‐0.219	0.470	0.098	0.571
Evoked pain						
PPT masseter	‐0.089	0.901	‐0.021	0.901	0.164	0.901
Jaw function						
MUO	0.109	0.516	‐0.185	0.516	0.266	0.351
JFLS	‐0.012	0.943	‐0.054	0.943	0.076	0.943

Note

*ρ* = Spearman's correlation coefficient; Hochberg's adjusted P‐values < 0.05 were considered as statistically significant.

Abbreviations: BMI, body mass index; CPI, characteristic pain intensity; JFLS, Jaw Functional Limitation Scale; MUO, maximal unassisted opening; PPT, pressure pain threshold; SODa, extracellular superoxide dismutase activity; TAC, total antioxidative capacity; TE, Trolox equivalents; TOS, total oxidant status.

## 4 DISCUSSION

This study confirmed that oxidative stress markers can be effectively measured in the saliva. Moreover, the main result of our study showed that the TAC levels were higher in TMDM, whereas TOS levels were lower, compared to healthy controls. In addition, no correlations between clinical characteristics of TMDM and the markers of oxidative stress were found. Thus, the hypothesis that oxidative stress markers could be dysregulated in TMDM appears to be confirmed.

Saliva has been proposed as a useful biofluid for assessing biomolecular profiles of several orofacial disorders, including TMD.^24^, ^25^, ^26^, ^27^, ^28^, ^29^ We have previously analysed the levels of pain‐associated markers, and the proteomic profile of plasma and saliva from healthy volunteers and individuals with TMDM.^18^, ^24^, ^30^ These studies suggest that stimulated whole saliva is a valuable medium to measure markers of pain in these pathologies. The proteomic analysis revealed several proteins associated with the immune system, stress response and metabolism that were dysregulated in TMDM.^18^ Our present study confirms that this is also the case for oxidative stress markers.

The involvement of oxidative stress has previously been suggested in TMD pathogenesis.^9^, ^31^, ^32^ For example, muscle levels of F2‐isoprostane were higher in a combined group of individuals with painful disc displacement and myalgia and were correlated to pain level and mechanical sensitivity.^34^ However, only few previous studies have investigated oxidative stress markers in saliva,^13^, ^15^, ^16^, ^33^ and none have focused on TMDM as main diagnosis.

Some studies have investigated TAC levels in TMD, but they have shown conflicting results in relation to our findings. Although we studied stimulated whole saliva, Vrbanovic et al ^15^ recently found that TAC levels were higher also in unstimulated whole saliva from a combined group of individuals with myofascial pain and painful disc displacement. On the contrary, Rodríguez de Sotillo et al ^13^ reported that TAC levels from unstimulated whole saliva from individuals with myalgia and arthralgia tended to be lower than in healthy controls. Almeida et al ^16^ supported their findings showing that individuals with arthralgia and/or myofascial pain with limited opening presented reduced TAC levels even in stimulated whole saliva compared to healthy subjects. In addition, in our proteomic study, performed in a subgroup of the samples used in the present investigation, levels of antioxidant proteins thioredoxin and S100‐A8 were lower as opposed to the increased TAC.^18^ These diverging results suggest that the collection method for whole saliva is not relevant for the quantification of oxidative stress markers and that antioxidant activity can be higher even when the number of antioxidants seems to be lower (at least at proteomic level).

Another factor that could have influenced the contrasting TAC results is that most previous articles mainly included individuals with TMJ diseases (eg painful disc disorders)^13^, ^15^, ^16^, ^34^; whereas we included individuals with myogenous TMDM as the main complaint. Regarding the diagnostic criteria, both the study by Rodríguez de Sotillo et al^13^ and ours used the DC/TMD; but they included individuals with myalgia and we mostly assessed individuals with myofascial pain. In contrast, the previous studies that evaluated myofascial pain, such as Almeida et al^16^ and Vrbanovic et al^15^ used the older RDC/TMD criteria for the classification—in which the myofascial pain category is more similar to the myalgia category found in DC/TMD.^6^, ^35^ Nevertheless, the impact of TMDM sub‐diagnoses on the measurement of oxidative stress markers is unclear, and we did not find any significant differences between them. Our results also suggest that the presence of TMJ arthralgia does not have a significant influence on oxidative stress markers in TMDM. But these should be carefully interpreted since these analyses might be underpowered because of the small sample sizes of the subgroups.

Another reason for the higher TAC levels in both our study and the study by Vrbanovic et al^15^ could be the chronicity of the disease. In the present study, the TMDM group had a median pain duration of five years (Table 1), suggesting that adaptation to oxidative stress might have occurred. This adaptation or hormesis occurs when mammalian cells respond to oxidative stressors by modulating antioxidative enzyme activity, and induce the clearance of these molecules.^36^, ^37^, ^38^ A study showed that longer exposure to a low level of oxidative agents might enhance this adaptative response in vitro and in vivo.^39^ In TMD, the relationship between chronicity and antioxidation was proposed by Vrbanovic et al^15^ as they included individuals with at least six months of pain duration. Other studies that showed opposite results did not clarify whether they included individuals with long‐term pain.^13^, ^16^

Regarding TOS, studies have reported higher overall oxidative status in TMD compared to healthy controls.^13^, ^40^ However, our results showed the opposite, that is, that the oxidant status was lower in individuals with TMDM (Figure 2A). This might be due to the high antioxidation found in the samples or to the chronicity, as reported for other pain‐related molecules (eg cortisol is sometimes reduced in chronic pain).^41^, ^42^ In the same line, Atanackovic et al^43^ showed that psychological stressors could suppress the formation of ROS. Thus, the lower TOS in our study could relate to the higher psychological stress levels compared to controls (Table 1).

In this investigation, no correlations were found between markers of oxidative stress and clinical characteristics, for example, pain, jaw function or psychological stress. Other studies have described that oxidative stress markers could be associated with strength loss and chronic muscle fatigue, rather than pain.^44^, ^45^, ^46^ However, specifically in the orofacial region, studies showed that TAC levels were positively correlated to MUO and were reduced in individuals with functional limitation.^31^, ^34^ Moreover, other studies found associations between the pain intensity and the dysregulation of oxidative stress markers.^13^, ^15^ The reasons behind the lack of correlations in our investigation are unclear, but the selection of TMDM as a main diagnosis cannot be discarded.

This is the first study that has investigated oxidative stress markers in saliva of TMDM also considering the sub‐diagnoses. Although there were some differences in TOS and TAC between individuals with myalgia and myofascial pain, these were non‐significant. As the main aim of the study was to compare TMDM with healthy controls, the study probably was underpowered for this sub‐analysis. The same might refer to the comparison of individuals with or without arthralgia. Exploring differences in these markers between TMDM subgroups could be an interesting topic for future research.

The main strength of this article is the exhaustive inclusion and exclusion criteria applied on the recruitment of both individuals with TMDM and controls. Another strength of the study is that blinding was performed during the sample analyses. Further, age and BMI were assessed to analyse their possible associations with the markers of oxidative stress as described in other investigations.^40^, ^47^ For instance, one study showed that in saliva of healthy subjects, the oxidation balance is age‐dependent.^48^ Moreover, another article showed that oxidative stress markers are impaired in obese young individuals compared to non‐overweight volunteers.^49^ In our study, there was no difference between groups regarding these factors (BMI and age), all participants were within normal weight range, and no correlations were found between them and oxidative stress markers.

A limitation of the present study is the cross‐sectional design, which did not allow for the description of causal interactions between pain, function and changes in oxidative stress markers over time.

In conclusion, in this study, the total salivary levels of oxidation seemed to be lower in TMDM as there might be an activation of a compensatory antioxidative mechanism; the TMDM group had higher total antioxidation with a lower total oxidant status. As reported in other articles, these changes might be associated with the role of oxidative stress in the chronicity and progression of TMDM, or the action of other TMD‐related factors, such as psychological stress, in oxidative stress itself. These results are a step forward into understanding the possible pathophysiological mechanisms behind these diseases. However, longitudinal studies are necessary to clarify whether there is a causal and temporal interaction between oxidative stress and TMDM.

## CONFLICT OF INTEREST

The authors declare no conflicts of interests.

## AUTHOR CONTRIBUTIONS

The experimental design and saliva testing procedures were performed by BG, HJ, ME and VIM. The recruitment and diagnosis of participants as well as the collection of samples were done by HJ. The statistical analyses were done by VIM. The first manuscript and figures were performed by VIM, and the consequent reviewing and editing were done by BG, HJ, ME and VIM. The supervision of the project was executed by ME.
